# Tissue factor activity of SW-480 human colon adenocarcinoma cells is modulated by thrombin and protein kinase C activation.

**DOI:** 10.1038/bjc.1998.640

**Published:** 1998-11

**Authors:** H. S. Chiang, R. S. Yang, S. W. Lin, T. F. Huang

**Affiliations:** Pharmacological Institute, College of Medicine, National Taiwan University, Taipei.

## Abstract

**Images:**


British Journal of Cancer (1998) 78(9), 1121–1127
© 1998 Cancer Research Campaign

### Tissue factor activity of SW-480 human colon adenocarcinoma cells is modulated by thrombin and protein kinase C activation

**H-S Chiang[1], R-S Yang[2], S-W Lin[3] and T-F Huang[1]**

[1]Pharmacological Institute, [2]Department of Orthopaedics and [3]Graduate Institute of Medical Technology, College of Medicine, National Taiwan University, No. 1, Sec. 1, Jen-Ai Rd, Taipei, Taiwan

**Summary** Expression of tissue factor (TF), a cellular initiator of the extrinsic coagulation cascade, is a feature of many malignant tumours and is intimately involved in the process of metastasis. SW-480 human colon adenocarcinoma cells responded to thrombin (1 U $ml^{-1}$) or phorbol 12-myristate 13-acetate (PMA, 0.1 μM) with a 6.0-fold and a 7.7-fold increase in their procoagulant activity (PCA), respectively, after 4–6 h incubation in serum-free medium. The thrombin-enhanced PCA was significantly inhibited by complexing of thrombin with hirudin, or by serine protease inhibition with 3,4-dichloroisocoumarin. Both effects of thrombin and PMA on PCA in SW-480 cells were blocked by pretreatment of cells with cycloheximide or actinomycin D, indicating that the response required de novo protein and RNA synthesis. The thrombin-enhanced PCA depended on the activation of protein kinase C (PKC) as it was diminished by staurosporine and calphostin C. Moreover, stimulation of SW-480 cells by thrombin or PMA led to a significant increase in TF mRNA within 3 h as measured by the reverse-transcription PCR method, which was also dependent on the activation of PKC. The unaltered decay rate of thrombin-enhanced TF mRNA, evaluated after the addition of staurosporine, suggested that its inhibitory effect occurred at a transcription level. Our data suggest that thrombin enhances TF gene expression and protein synthesis in tumour cells in vitro via PKC activation. The induction of TF expression in tumour cells by thrombin indicates that tumour-associated PCA might have a positive-feedback effect on in vivo local propagation of thrombus by thrombin formation.

**Keywords:** colon adenocarcinoma; tissue factor; thrombin; protein kinase C; mRNA synthesis; metastasis

Tissue factor (TF), a 47-kDa integral membrane glycoprotein, is an essential co-factor for factor VII/VIIa, which triggers the cell-surface assembly of coagulation protease cascade, finally leading to formation and deposition of fibrin (Altieri and Edgington, 1988). The constitutively expressed TF is preferentially detectable in the extravascular cells of many tissues (Drake et al, 1989; Fleck et al, 1990) such as epidermis, cerebral cortex, kidney glomeruli, mucosal epithelial layers and adventitia of vessels. Within the vasculature, endothelium lacking TF under physiological conditions can be stimulated by endotoxins, thrombin or cytokines to start de novo TF synthesis (Brox et al, 1984; Edgington et al, 1991; Tijburg et al, 1991). Similar results have been found in monocytes, in which TF synthesis is stimulated by a variety of inflammatory mediators and antigen-specific cellular immune responses (Edgington et al, 1991). However, little is known about the regulatory mechanisms of constitutive TF expression detected in the cells of certain malignant tumours (Callander et al, 1992; Chiang et al, 1994, 1995).

It has been known that most cancer patients manifest signs of hypercoagulability and some develop thromboembolic disease (Bick, 1992; Rickles et al, 1992). There is evidence that systemic or local activation of blood coagulation promotes metastasis, whereas inhibition of the blood-clotting cascade favours the host and diminishes metastatic spread (Honn and Sloane, 1984). Therefore, it has been suggested that an efficient metastasis of tumour cells is possibly dependent on the plasma coagulation cascades. Tumour-associated procoagulant activity (PCA) is thought to lead to local thrombin generation and peritumour fibrin formation in the presence of an intact local coagulation pathway, which has been considered a potentially important characteristic in the malignant state (Zacharski et al, 1990). Although an alternative pathway exists to activate coagulation, including a tumour cell proteinase-designed cancer procoagulant (Gordon et al, 1975), the in vivo activation of this proteinase system predominantly proceeds via the TF pathway (Davie et al, 1991) arising from constitutive TF expression in a variety of tumour cell lines. Indeed, the role of TF in promoting metastasis has been shown in a melanoma metastasis model (Fidler, 1986; Mueller et al, 1992) in which melanoma cell lines expressing high levels of TF exhibited metastasis strongly, whereas the metastatic potential of the cell lines could be inhibited by treatment with an anti-TF MAb that blocked its PCA. These results suggested that certain component(s) of the coagulation cascade might mediate the metastasis effect of TF expression on tumour cells. Thrombin, a pluripotent bioregulatory serine proteinase, has been reported to enhance the metastatic phenotype of mammary tumour cells by inducing their proliferative response (Medrano et al, 1987) and found to be a potent mitogen for tumour cells (Bruhn and Zurborn 1983). Adhesion of tumour cells to platelets (Nierodzik et al, 1991, 1992), endothelium (Wojtukiewicz et al, 1993) and the subendothelial matrix (Klepfish et al, 1993) has also been reported to be stimulated by thrombin. Furthermore, it has been reported that

*Received 8 October 1997*
*Revised 28 February 1998*
*Accepted 2 April 1998*

*Correspondence to:* T-F Huang thrombin-treated tumour cells markedly enhanced pulmonary metastasis (Nierodzik et al, 1991, 1992). Our previous study showed that thrombin enhanced the adhesive and migratory activities of SW-480 cells via up-regulated $\beta_3$ integrin expression (Chiang et al, 1996). In addition, thrombin was shown to mediate tumour cell-induced platelet aggregation (TCIPA) owing to TF activity expression on SW-480 cells (Chiang et al, 1994), which might be important for successful metastasis to occur (Cavanaugh et al, 1988).

The aim of our present study is to explore the possible regulation of TF expression in SW-480 human adenocarcinoma cells. Thrombin was found to induce TF mRNA and protein synthesis in SW-480 cells rapidly and markedly, which is dependent on activation of protein kinase C (PKC). The induction of TF expression in tumour cells by thrombin suggests that tumour-associated PCA might have a positive-feedback effect on in vivo local propagation of thrombus by thrombin formation.

**Figure 1** Bar graph of time course of thrombin on procoagulant TF activity. SW-480 cells were incubated with either serum-free DMEM or together with thrombin (1 U ml$^{-1}$) for 48 h at 37°C. Cell monolayers were harvested at the time indicated, and then disrupted and assayed for PCA, as described in Materials and methods. Data of thrombin treatment are presented as mean ± s.e.m. ($n$ = 3). □, Thrombin-enhanced PCA; ■, constitutive PCA

### MATERIALS AND METHODS

### Materials

SW-480 human colon adenocarcinoma cells were provided by the Department of Bacteriology, College of Medicine, National Taiwan University. Human thrombin (3000 NIH units mg$^{-1}$), hirudin (grade IV from leeches), 3,4-dichloroisocoumarin (3,4-DCI), cycloheximide, actinomycin D and fibronectin (from bovine plasma), and phospholipase C (*Baccillus cereus*) were obtained from Sigma, St Louis, MO, USA. Thrombin–hirudin or thrombin–serine protease inhibitor complex was formed by incubation for 30 min at 37°C of equimolar concentrations of human thrombin with either inhibitor (hirudin, 5 U ml$^{-1}$; 3,4-DCI, 0.1 mM). The thrombin–hirudin complex exhibited no fibrinogen clotting activity. Staurosporine was obtained from Biomol Research Laboratories, PA, USA. Calphostin C (isolated from *Cladosporium cladosporioides*) was from Research Biochemicals International, MA, USA. Goat anti-mouse IgG-FITC was from Boehringer, Mannheim, Germany. Monoclonal antibody (MAb) A135 raised against human TF was obtained from Enzyme Research Labs.

### Cell culture

SW-480 human colon adenocarcinoma cells were cultured in a humidified atmosphere of 5% carbon dioxide and 95% air in a mixture of DMEM tissue culture medium supplemented with 10% heat-inactivated fetal calf serum (FCS), 2 mM L-glutamine, penicillin (100 U ml$^{-1}$) and streptomycin (100 mg ml$^{-1}$). Confluent monolayers were passaged from culture flasks with brief treatment of 0.1% trypsin–1 mM EDTA.

### Treatment of SW-480 cells

For stimulation, cells were harvested from confluent monolayer cultures after detachment by trypsin–EDTA and seeded in 25-cm$^2$ culture flasks (Costar, MA, USA.) with a complete medium at a concentration of 1 × 10$^5$ cells. After 3 days, cell monolayers were washed three times with PBS and the cell culture was continued by incubation with serum-free DMEM containing various concentrations of thrombin, thrombin–hirudin or thrombin–serine proteinase complex. Conditioned medium was removed and tumour cells were harvested mechanically at the desired culture intervals with a cell scraper (Costar), washed three times with phosphate-buffered saline (PBS) and adjusted to 5 × 10$^4$ cells ml$^{-1}$. Viability of tumour cells was 90–95% as examined by Trypan blue exclusion. The cell suspension (1 ml) was pelleted by centrifugation and the supernatant was decanted; the pellets were then frozen at –20°C for further experiment.

### Measurement of procoagulant activity

The procoagulant activity (PCA) of cell preparations was measured by plasma recalcification time (Chiang et al, 1994). After thawing, the cell pellets were lysed with 30 µl of 16 mM octyl-β-D-glycopyranoside at 37°C for 10 min and diluted in 70 µl of 25 mM Hepes–saline (Flossel et al, 1992). Platelet-poor plasma was prepared from whole blood collected from healthy human volunteers and mixed with 3.8% (w/v) sodium citrate (9:1, v/v). In the coagulation assay, 100 µl of normal citrated plasma was incubated with 100 µl of the cell lysate for 2 min at 37°C. Thereafter, 100 µl of prewarmed 25 mM calcium chloride was added, and the plasma clotting time was determined by a fibrometer (Coag-a-mate, Organon Teknika, NC, USA). The time recorded was converted to milliunits (mU) per 5 × 10$^4$ cells of PCA by reference to a standard curve constructed with serial dilutions of a commercial thromboplastin (Simplastin, Organon Teknika, USA). The amount of thromboplastin required to produce a clotting time of 17 s was arbitarily assigned as one PCA unit. To determine the role of the TF–factor VII/VIIa pathway in the PCA, normal human plasma was replaced in some experiments by a factor VII-deficient plasma. In other control experiments, cells were incubated with phospholipase C (1 U ml$^{-1}$) at 37°C for 15 min before the clotting assay.

### Flow cytometric analysis

Flow cytometric studies were performed to quantify surface expression of TF antigen (Chiang et al, 1996). SW-480 cells were stimulated with thrombin (1 U ml$^{-1}$) for 4 h at 37°C, detached

© Cancer Research Campaign 1998

**Figure 2** Effect of various concentrations of thrombin (**A**) and PMA (**B**) on procoagulant activity. Thrombin and PMA at the final concentrations shown were added to SW-480 cells in serum-free DMEM for 4 h at 37°C. Cell monolayers were then disrupted and assayed for PCA, as described in Materials and methods. Data are presented as mean ± s.e.m. ($n$ = 4)

**Figure 3** Effect of hirudin as well as inhibitors of serine proteinase, protein synthesis and RNA synthesis on thrombin-enhanced TF activity of SW-480 cells. SW-480 cells were stimulated with optimal dose of thrombin (1 U $ml^{-1}$), thrombin–hirudin or thrombin–3,4-DCI complex (each complex contained 1 U $ml^{-1}$ thrombin) for 4 h at 37°C, and then assayed for PCA. In other experiments cells were incubated with various concentrations of cycloheximide or actinomycin D for 20 min at room temperature before the addition of thrombin (1 U $ml^{-1}$) for 4 h at 37°C. Data are presented as mean ± s.e.m. ($n$ = 4)

(using 0.5 mM EDTA), washed and then suspended in 500 μl of PBS containing $10^6$ cells per sample. Following washing, the cells were fixed with 2.7% paraformaldehyde for 10 min, blocked with normal goat serum (1:2) for 25 min and labelled with human anti-TF MAb (A135, 20 μg $ml^{-1}$) for 1 h. After washing, the cells were relabelled with goat anti-mouse IgG-FITC. FITC signals were detected and digitized in logarithmic configuration. A total of 10 000 cells were counted per experimental group and data were analysed by using a FACScan (Becton Dickinson). The control fluorescence intensity was obtained with cell suspension in which primary antibody was replaced with non-immune IgG. All experiments were repeated at least four times.

### RNA isolation, reverse transcription and PCR for TF mRNA

For total RNA preparation, confluent SW-480 cell monolayers in 25-$cm^2$ flasks were incubated in serum-free DMEM for various time intervals with thrombin (1 U $ml^{-1}$) or PMA (0.1 μM). RNA was extracted by using the Trizol reagent (Gibco BRL, NY, USA): 0.2 ml of chloroform and 0.5 ml of isopropyl alcohol per 1 ml of Trizol reagent were added for phase separation and RNA precipitation respectively, and centrifuged at 12 000 ***g*** for 10 min at 4°C. The RNA pellet was extracted by ethanol, dried, dissolved in 25 μl of water and the concentration of RNA determined by absorbance at 260 nm. Purified RNA samples were stored at −20°C.

An appropriate amount of RNA (2–3 μg) was mixed with 2.5 μM oligo(dT) primer, hexamer (0.9 μg $μl^{-1}$), heated to 70°C and then quickly chilled on ice. Each of the above RNA mixtures was for reverse transcription by incubating the mixture at 42°C for 1 h with 200 units of reverse transcriptase (MMLV Superscript II system: BRL, Bethesda, MD, USA) in a reaction buffer containing 50 mM Tris-HCl (pH 8.3), 75 mM potassium chloride, 3 mM magnesium chloride, 10 mM DTT, 500 μM of each of the four dNTPs and 40 U of rRNasin (Promega). After synthesis of the first-strain cDNA, aliquots of the RT mixtures were subjected to regular PCR (35 rounds of 94°C for 1 min, 55°C for 2 min and 72°C for 2 min) using appropriate primer pairs, TF186 and TFD. The sequences for primers are as follows: TF186 5′ GGAGAAAACTACTGTTTCAGTGTTCAAGCAGT-GATT 3′, corresponding to nucleotides 739–774; and TFD, 5′ AATATAG-CATTTGCAGTAGCTCCAACAGTGCTTCC 3′, corresponding to nucleotides 1023–989. The sequences for internal control, Gβ-like primers, are: Gβa20, 5′ GTATGGAACCTGGCTAACTG 3′, corresponding to nucleotides 620–640; and Gβc20, 5′ TAGGGCT-GAAGCACAGGGCG 3′, corresponding to nucleotides 825–806. The amplified samples were electrophoresed on a 2% agarose gel, followed by staining with ethidium bromide, and photographed with Polaroid 667 negative film. RNA quantification was achieved by scanning analysis of the bands using UVP Image Store 2000 (CA, USA) and the NIH IMAGE program.

**Figure 4** Effect of staurosporine and calphostin C on thrombin-enhanced TF activity. SW-480 cells were incubated with various concentrations of staurosporine and calphostin C for 20 min at room temperature, then stimulated with thrombin (1 U ml$^{-1}$) for 4 h at 37°C. Data are presented as mean ± s.e.m. ($n = 4$)

**Figure 5** Effect of thrombin and PMA on TF mRNA levels in SW-480 cells as revealed by RT-PCR analysis Total cellular RNA (2–3 µg) was reverse transcribed and then amplified using TF- and Gβ-actin-specific primers. The molecular size was determined using *Hae*III DNA molecular marker. PCR products of TF and Gβ-actin mRNA were from SW-480 cells cultured for 1, 3, 5 and 7 h in serum-free DMEM alone (lanes 1–4 respectively), in the presence of 1 U ml$^{-1}$ thrombin for 1, 3, 5 and 7 h (lanes 5–8 respectively), or 0.1 µM PMA for 1, 3, 5 and 7 h (lanes 9–12 respectively).

**Table 1** Densitometric scan of 2.8-kb TF mRNA

| Treatment[a] | TF mRNA | |
|---|---|---|
| | Densitometric units[b] | Percentage[c] |
| Thrombin alone | 1787 | 100 |
| + staurosporine 0.25 µM | 983 | 55 |
| + staurosporine 0.5 µM | 822 | 46 |
| + staurosporine 1 µM | 393 | 22 |
| Thrombin alone | 1911 | 100 |
| + calphostin C 1.25 µM | 841 | 44 |
| + calphostin C 2.5 µM | 382 | 20 |
| + calphostin C 5 µM | 344 | 18 |

[a]SW-480 cells were incubated in the presence of thrombin (1 U ml$^{-1}$) with or without PKC inhibitors at the indicated concentrations for 4 h, as described in Materials and methods. RT-PCRs were prepared with total cellular RNA (2–3 µg) and amplified simultaneously or consecutively with primers for TF and Gβ-like. [b]Arbitrary units. Values were corrected relative to the Gβ-like control. [c]Percentage of values obtained without inhibitor.

### Transcript stability analysis

The effect of staurosporine on TF mRNA stability was examined by inhibiting mRNA transcription with actinomycin D. After incubating the cells with thrombin for 2 h, actinomycin D (5 µM) was added with or without staurosporine (1 µM) and the disappearance of TF mRNA was analysed by RT-PCR. Values were calculated relative to the Gβ-like control.

### RESULTS

### Constitutive TF expression on SW-480 cells

In the presence of serum-free DMEM without the addition of thrombin, SW-480 cells harvested from subconfluent monolayer cultures at various time periods showed neither the enhanced constitutive PCA measured by clotting assay (Figure 1) nor the molecular TF expression assayed by flow cytometric analysis (data not shown). No PCA was generated when factor VII-deficient plasma instead of normal plasma was used in the clotting assay.

### Enhancement of PCA by thrombin and phorbol ester on SW-480 cells

To study the time course of thrombin-stimulated TF expression, SW-480 cells were cultured with thrombin (1 U ml$^{-1}$) over a period of 0–48 h, then PCA of these treated cells was analysed. Figure 1 shows that a significant increment of PCA was observed after 2-h-incubation, reaching the maximum at 4–6 h, gradually declining to the basal value within 48 h. During a 4-h incubation, stimulation of SW-480 cells with various concentrations of thrombin exhibited a bell-shaped dose–response curve in enhancing PCA with a peak at 1 U ml$^{-1}$ (Figure 2A). The maximal effect was approximately sixfold. After the treatment of 1 U ml$^{-1}$ thrombin for 4 h at 37°C, an increased expression of TF antigen within the cell population was found by flow cytometric analysis (data not shown).

It has been reported that the activation of protein kinase C (PKC) is involved in tumour metastasis and induction of TF expression in monocytes (Ternisien et al, 1993) and epithelial cells (Terry et al, 1996). Therefore we examined the effect of phorbol 12-myristate 13-acetate (PMA), a phorbol ester directly stimulating PKC, on SW-480 TF expression. As shown in Figure 2B, PMA exhibited a bell-shaped dose–response curve in increasing PCA with a maximal 7.7-fold effect at 0.1 µM. On the other hand, neither thrombin- nor PMA-stimulated SW-480 cells were capable of inducing PCA in factor VII-deficient plasma, and the enhanced PCA was reconfirmed as increased expression of TF by the observation that the enhanced PCA was lost in the presence of phospholipase C (1 U ml$^{-1}$).

### Effects of thrombin inhibitors and metabolic inhibitors on thrombin-enhanced PCA

Figure 3 shows results of various inhibitors on thrombin-enhanced TF activity. When thrombin was coupled with hirudin, a specific

© Cancer Research Campaign 1998

**Figure 6** Effect of staurosporine on the stability of thrombin-enhanced TF mRNA. SW-480 cells were incubated with thrombin (1 U ml$^{-1}$) for 2 h to enhance TF mRNA. Actinomycin D (5 μM) was then added to all samples and further incubation was performed in the presence or in the absence of staurosporine (1 μM). Total mRNA was extracted at the various time intervals indicated, and TF mRNA was analysed by RT-PCR. The values shown represent the percentage of maximal TF mRNA obtained just after the addition of actinomycin D. Data are presented as average value (*n* = 2) □, Actinomycin D; ▨, actinomycin D + staurosporine thrombin inhibitor, a profound inhibition was obtained when compared with thrombin alone. This suggests that an intact thrombin molecule is apparently required for enhancing TF activity of SW-480 cells. A similar effect was found by pretreatment of thrombin with 3,4-DCI, an irreversible serine protease inhibitor, indicating that the catalytic site of serine residue is required for the enhancing effect of thrombin. In addition, preincubation of SW-480 cells with either actinomycin D or cycloheximide abolished thrombin- or PMA (data not shown)-enhanced PCA, further suggesting that thrombin-enhanced TF activity requires mRNA and de novo protein synthesis.

### Effects of PKC inhibitors on thrombin-enhanced PCA

The effects of PKC inhibitors were used to examine whether PKC is implicated in thrombin-enhanced TF activity. As shown in Figure 4, pretreatment of SW-480 cells with either staurosporine (0.04–1 μM), a potent but relatively less selective PKC inhibitor, or calphostin C (0.2–5 μM), a selective PKC inhibitor, blocked thrombin-enhanced PCA in a dose-dependent manner. Maximal inhibition was observed to a level of 50–75% of control (i.e. unstimulated) PCA, suggesting that PKC activation is a key event in thrombin effect.

### Effect of thrombin and PMA on TF mRNA levels

Induction of TF activity could be due to an increase in *TF* gene expression or an increase in the rate of initiation of coagulation by the existing TF protein. Therefore, we determined whether changes in TF mRNA levels could account for the observed induction of TF activity. TF mRNA was quantified using the reverse transcription-PCR method. The products of the reverse transcription-PCR were analysed on a 2% agarose gel and stained with ethidium bromide. When 2–3 μg of total RNA of untreated SW-480 cells were analysed, TF mRNA was constutively detectable as a single band having an approximate size of 0.28 kb, corresponding to the size (285 bp) predicted from the published sequence (Figure 5). The identity was further established by the cleavage of the 280 bp band by *Eco*RI into approximately 180- and 100-bp fragments. Whereas the contents of Gβ-actin mRNA remained unchanged, TF mRNA levels in the control (unstimulated) cells decreased in a time-dependent manner after replacement of serum-free medium. However, following stimulation with thrombin (1 U ml$^{-1}$) or PMA (0.1 μM), TF mRNA increased greatly at 3 h incubation and decreased at 5–7 h (Figure 5). Quantification by densitometry of the amplified TF species and comparison with the Gβ-actin species demonstrated that TF mRNA was increased 1.9- and 2.1-fold by thrombin and PMA respectively, at 3 h incubation.

### Effect of thrombin inhibitors on thrombin-enhanced TF mRNA levels

We compared TF mRNA levels in SW-480 cells stimulated with thrombin, hirudin coupled-thrombin or 3,4-DCI-treated thrombin. In contrast to thrombin-enhanced TF mRNA, either hirudin–thrombin or 3,4-DCI–thrombin used at the same concentration as active thrombin showed a significantly decreased TF mRNA expression in SW-480 cells (0.4- and 0.5-fold respectively).

### Effect of PKC inhibitors on thrombin-enhanced TF mRNA levels

PKC inhibitors were used in conjunction with thrombin to identify the signal transduction pathways involved in the regulation of TF gene expression. SW-480 cells treated with increasing concentrations of either staurosporine (0.25–1 μM) or calphostin C (1.25–5 μM) showed a dose-dependent decrease in the production of TF mRNA. Table 1 showed the quantitative results of the inhibitory effects of staurosporine and calphostin C on thrombin-enhanced TF mRNA expression in SW-480 cells.

### Effect of staurosporine on TF mRNA stability

After incubation with thrombin for 2 h, transcription was arrested by actinomycin D and further incubation was performed with or without the addition of staurosporine (1 μM). The disappearance of TF mRNA with time was analysed by RT-PCR with scanning densitometry. As shown in Figure 6, the yield of mature 2.8-kb TF mRNA fell in a similar way in the presence or absence of staurosporine. A 50% decrease in TF mRNA was detected at about 1.5 h in both conditions, suggesting that the addition of staurosporine after the arrest of transcription did not modify the disappearance rate of TF mRNA.

### DISCUSSION

Experimental evidence suggests that blood coagulation is activated at the site of tumour cell lodgement and plays a role in the pathology of metastatic tumour growth. Procoagulant activity produced by tumour cells has been implicated in this process (Amirkhosravi et al, 1995). The common occurrence of activating blood coagulation in most cancer patients leads to the generation of thrombin; thus, it seems reasonable to explore whether thrombin has any direct influence in enhancing the process of tumour cell metastasis. Thrombin has been reported to favour metastatic spread of cancer by promoting cell migration, enhancing tumour-cell adhesion, stimulating secretion of autocrine

© Cancer Research Campaign 1998

growth factor or inducing neovascularization because of its role in TCIPA and fibrin formation (Nierodzik et al, 1991; Zacharski et al, 1990; Chiang et al, 1995). Our present study showed that thrombin enhanced PCA of SW-480 human colon adenocarcinoma cells by enhancing their TF expression, which may occur in vivo, potentiating the haematogenous metastatic ability of tumour cells.

Thrombin has previously been shown to elicit a variety of functional responses in human endothelial cell cultures, including induction of TF mRNA and protein synthesis in a time- and dose-dependent manner (Galdal et al, 1985; Bartha et al, 1993). A similar effect was also reported in cultured vascular smooth muscle cells (Taubman et al, 1993). The data presented here show that the PCA of SW-480 human colon adenocarcinoma cells was up-regulated by thrombin as well as by PMA. The thrombin-enhanced PCA was inhibited by phospholipase C and was not expressed when tested in factor VII-deficient plasma, thus confirming that the up-regulated PCA was due to the enhanced TF activity of SW-480 cells. This is consistent with the previous observation that close association with certain membrane phospholipids is essential for the functional activity of TF, as phospholipase C treatment destroyed procoagulant activity of TF in monocytes and renal glomeruli (Otnaess et al, 1972; Kornberg et al, 1994; Tipping et al, 1998). The time- and dose-dependent manner of thrombin in enhancing PCA suggests a thrombin receptor-mediated mode of action, but the identification of the receptors and their role in mediating thrombin-enhanced TF activity are not known. However, we found that the active site of serine residue in thrombin molecule was a prerequisite for its full expression on TF activity, as pretreatment of thrombin with hirudin or 3,4-DCI resulted in a lower potency than the native thrombin. In addition, recent data suggested that the effect of thrombin on inducing TF mRNA levels in endothelial cells might be at least partly mediated through 'tethered ligand' thrombin receptor (Deguchi et al, 1997; Bartha et al, 1993), as recently reported by Wojtujiewicz et al (1995) with human colon adenocarcinoma (colon A) and by Fisher et al (1995) with M24met melanoma cells.

Thrombin- and PMA-enhanced TF activity showed a bell-shaped dose–response curve with optimal concentration at 1 U $ml^{-1}$ and 0.1 $\mu$M respectively. At higher concentrations, both agonists enhanced less TF activity. In addition, the same profile was found for TF antigen (data not shown). The mechanism by which high concentrations of both agonists induced a decrease in TF activity and antigen is unclear, although PMA has been reported to shed TF from the cell membrane (Brozna and Carson 1988). Several observations have suggested that PKC is involved in mediating the cellular effects of thrombin (Herrick-Davis et al, 1997; Gomez et al, 1988). To determine whether the effects involved PKC or other protein kinases, the inhibitors tested were staurosporine and calphostin C. Staurosporine interferes with the ATP-binding sites of PKC, whereas calphostin C appears to interfere with the phospholipid-binding regulatory site and is much more specific for PKC when used appropriately (Bruns et al, 1991). Both inhibitors blocked the thrombin-enhanced TF activity in a concentration-dependent manner and negatively affected the TF mRNA content, indicating that exposure of SW-480 to thrombin activates PKC, and that this activation is involved in the induction of TF gene expression, thereby leading to the synthesis of TF by SW-480 cells. Furthermore, treatment of the cells with cycloheximide and actinomycin D completely inhibited the TF activity enhanced by both agents, suggesting that de novo synthesis of protein and mRNA is required. The mechanism whereby PKC activation leads to de novo synthesis of TF protein and mRNA remains to be elucidated. PKC represents a family of serine/threonine protein kinases that provide regulatory functions in intracellular signal transduction and are implicated in tumour growth, promotion and differentiation as well as oncogene activation and carcinogenesis (Liu et al, 1992).

To date only initial investigations have been made into the modulation of constitutive TF expression in other systems. In COS-7 cells, expressing high levels of TF in culture, a novel serum response element has been found in the TF promoter region (Mackman et al, 1990). In general, the TF gene has been classified as an immediate early gene responsive to serum, purified growth factors or certain hormones, which has suggested that TF may participate in biological processes other than haemostasis, including cell proliferation, inflammatory responses, wound healing and the effector limb of the immune system. Whereas the PKC inhibitor-induced decrease in TF mRNA supports the possibility of a decrease in TF transcription, the method of RNA analysis used could not distinguish between an effect on the transcription rate and/or an effect on TF mRNA stability. As TF mRNA stability was unaffected by the addition of staurosporine after arrest of transcription by actinomycin D, a post-transcriptional mechanism induced by this inhibitor is unlikely to be involved in the decrease in TF mRNA levels. These results are in accordance with those obtained with other genes, stating that stability is unaffected by treatment with H7, a specific PKC inhibitor. In contrast, the decay rate of tumour necrosis factor $\alpha$ mRNA is greatly enhanced by PKC inhibitor, which significantly increases the rate of poly(A) removal from tumour necrosis factor $\alpha$ mRNA, thus facilitating its degradation (Lieberman et al, 1992).

In conclusion, our results suggest that thrombin-enhanced TF expression in SW-480 human colon adenocarcinoma cells involves PKC activation. Staurosporine-suppressed TF mRNA was not associated with an apparent alteration in its stability, suggesting that the effect is mainly attributable to a decrease in transcription. As the enhancement of TF expression in tumour cells by thrombin may contribute to the haematogenous phase of metastasis, further investigation of the intracellular signalling pathways involved in thrombin-enhanced TF synthesis will open up the possibility of modulating this response.

### ACKNOWLEDGEMENTS

The authors would like to thank Dr Wen-Hwa Lee for supplying Gβ-like primers and Miss Wen-Chun Chang for providing excellent technical assistance. This work was financially supported by a grant from the National Science Council of Taiwan (NSC-85-2331-B002-267).

### REFERENCES

Amirkhosravi M and Francis JL (1995) Coagulation activation by MC28 fibrosarcoma cells facilitates lung tumor formation. *Thromb Haemost* **73**: 59–65

Altieri DC and Edgington TS (1988) Sequential receptor cascade for coagulation proteins on monocytes. *J Biol Chem* **264**: 2969–2979

Bartha K, Brisson C, Archipoff G, de La Salle C, Lanza F, Cazenave J-P and Beretz A (1993) Thrombin regulates tissue factor and thrombomodulin mRNA levels and activities in human saphenous vein endothelial cells by distinct mechanisms. *J Biol Chem* **268**: 421–429

Bick RL (1992) Coagulation abnormalities in malignancy – a review. *Semin Thromb Hemostasis* **18**: 353–372

© Cancer Research Campaign 1998

Brox JH, Osterud B, Bjorklid E and Fenton II JW (1984) Production and availability of thromboplastin in endothelial cells: the effects of thrombin, endotoxin and platelets. *Br J Haematol* **57**: 239–246
Brozna JP and Carson SD (1988) Monocyte-associated tissue factor is suppressed by phorbol myristate acetate. *Blood* **72**: 456–462
Bruhn HD and Zurborn KH (1983) Influences of clotting factors (thrombin, factor XIII) and fibronectin on the growth of tumor cells and leukemic cells *in vitro*. *Blut* **46**: 85–88
Bruns RF, Dean Miller F, Merriman RL, Howbert JJ, Heath WF, Kobayashi E, Takahashi I, Tamaoki T and Nakano H (1991) Inhibition of protein kinase C by calphostin C is light dependent. *Biochem Biophys Res Commun* **176**: 288–293
Callander NS, Varki N and Rao LVM (1992) Immunohistochemical identification of tissue factor in solid tumors. *Cancer* **70**: 1194–1201
Cavanaugh PG, Sloane BF and Honn KV (1988) Role of the coagulation system in tumor cell-induced platelet aggregation and metastasis. *Haemostasis* **18**: 37–46
Chiang HS, Swaim MW and Huang TF (1994) Characterization of platelet aggregation induced by human colon adenocarcinoma cells and its inhibition by snake venom peptides, trigramin and rhodostomin. *Br J Haematol* **87**: 325–331
Chiang HS, Yang RS and Huang TF (1996) Thrombin enhances the adhesive and migratory properties of human colon adenocarcinoma cells via increased $\beta_3$-integrin expression on tumor cell surface and their inhibition by snake venom peptide, rhodostomin. *Br J Cancer* **73**: 902–908
Chiang HS, Swaim MW and Huang TF (1995) Characterization of platelet aggregation induced by human breast carcinoma and its inhibition by snake venom peptides, trigramin and rhodostomin. *Breast Cancer Res Tr* **33**: 325–335
Davie EW, Fujikawa K and Kisiel W (1991) The coagulation cascade: initiation, maintenance and regulation. *Biochemistry* **30**: 10363–10370
Deguchi H, Takeya H, Wada H, Gabazza EC, Hayashi N, Urano H and Suzuki H (1997) Dilazep, and antiplatelet agent, inhibits tissue factor expression in endothelial cells and monocytes. *Blood* **90** (6): 2345–2356
Drake TA, Morrissey JH and Edgington TS (1989) Selective cellular expression of tissue factor in human tissues. *Am J Pathol* **134**: 1087–1097
Fidler IJ (1986) Rationale and methods for the use of nude mice to study the biology and therapy of human cancer metastasis. *Cancer Metastasis Rev* **5**: 29–49
Fischer EG, Ruf W and Mueller M (1995) Tissue-factor-initiated thrombin generation activates the signaling thrombin receptor on malignant melanoma cells. *Cancer Res* **55**: 1629–1632
Fleck RA, Rao LVM, Rapaport SI and Varki N (1990) Localization of human tissue factor antigen by immunostaining with monospecific, polyclonal anti-human tissue factor antibody. *Thromb Res* **59**: 421–437
Gordon SG, Franks JJ and Lewis B (1975) Cancer procoagulant A: a factor X activating procoagulant from malignant tissue. *Thromb Res* **6**: 127–137
Galdal KS, Lyberg T, Evensen SA, Nilsen E and Prydz H (1985) Thrombin induces thromboelastin synthesis in cultured vascular endothelial cells. *Thromb Haemost* **54**: 373–376
Herrick-Davis K, Camussi G, Bussolino F and Baglioni C (1991) Modulation of neurite outgrowth in neuroblastoma cells by protein kinase C and platelet-activity factor. *J Biol Chem* **266** (28): 18620–18625
Honn KV and Sloane BF (1984) *Hemostatic Mechanisms and Metastasis*. Boston: Martinus Nijhoff
Klepfish A, Greco MA and Karatkin S (1993) Thrombin stimulates melanoma tumor-cell binding to endothelial cells and subendothelial matrix. *I J Cancer* **53** (6): 978–982
Kornberg A, Blank M, Kaufman S and Shoenfeld Y (1994) Induction of tissue factor-like activity in monocytes by anti-cardiolipin antibodies. *J Immunol* **153**: 1328–1332
Lieberman AP, Pitha PM and Shin ML (1992) Ploy(A) removal is the kinase-regulated step in tumor necrosis factor mRNA decay. *J Biol Chem* **267**: 2123–2126
Mackman N, Fowler BJ, Edgington TS and Morrissey JH (1990) Human tissue factor gene: functional analysis for expression in COS-7 cells. *Proc Natl Acad Sci USA* **87**: 2254–2258
Medrano EE, Cafferata EGA and Larcher F (1987) Role of thrombin in the proliferative response of T-47D mammary tumor cells. *Exp Cell Res* **172**: 354–364
Mueller BM, Reisfeld RA and Edgington TS, Ruf W (1992) Expression of tissue factor by melanoma cells promotes efficient hematogenous metastasis. *Proc Natl Acad Sci USA* **89**: 11832–11836
Nierodzik ML, Kajumo F and Karpatkin S (1992) Effect of thrombin treatment of tumor cells on adhesion of tumor cells to platelets *in vitro* and tumor metastasis *in vivo*. *Cancer Res* **52**: 3267–3272
Nierodzik ML, Plotkin A, Kajumo F and Karpatkin (1991) Thrombin stimulates tumor–platelet adhesion *in vitro* and metastasis *in vivo*. *J Clin Invest* **87**: 229–236
Otnaess AB, Prydz H, Bjorklid E and Berre A (1972) Phospholipase C from *Bacillus cereus* and its use in studies of tissue thromboplastin. *Eur J Biochem* **27**: 238–243
Rickles FR, Levine M and Edwards RL (1992) Hemostatic alterations in cancer patients. *Cancer Metastasis Rev* **11**: 237–248
Taubman MB (1993) Tissue factor regulation in vascular smooth muscle: a summary of studies performed using in vivo and in vitro models. *Am J Cardiol* **72** (8): 55C–60C
Ternisen C, Ramani M, Ollivier V, Khechai F, Vu T, Hakim J and de Prost D (1993) Endotoxin-induced tissue factor in human monocytes is dependent upon protein kinase C activation. *Throm Haemostas* **70** (5): 800–806
Terry CM and Callahan KS (1996) Protein kinase C regulates cytokine-induced tissue-factor transcription and procoagulant activity in human endothelial cells. *J Lab Clin Med* **127** (1): 81–93
Tijburg PMN, Ryan J, Stern DM, Wollitzky B, Rimon S, Rimon A, Handley D, Nawroth P, Sixma JJ and de Groot PG (1991) Activation of the coagulation mechanism on tumor-necrosis factor-stimulated cultured endothelial cells and their extracellular matrix. *J Biol Chem* **266**: 12067–12074
Tipping PG, Dowling JP and Holdsworth SR (1998) Glomerular procoagulant activity in human proliferative glomerulonephritis. *J Clin Invest* **81**: 119–125
Wojtukiewicz MZ, Tang DG, Ben-Josef E, Renaud C, Walz DA and Honn KV (1995) Solid tumor cells express functional 'tethered ligand' thrombin receptor. *Cancer Res* **55**: 698–704
Zacharski LR, Memoli VA, Costantini V, Wojtukiewicz MZ and Ornstein DL (1990) Clotting factors in tumor tissue: implications for cancer therapy. *Blood Coagulat Fibrinol* **1**: 71–78

© Cancer Research Campaign 1998 


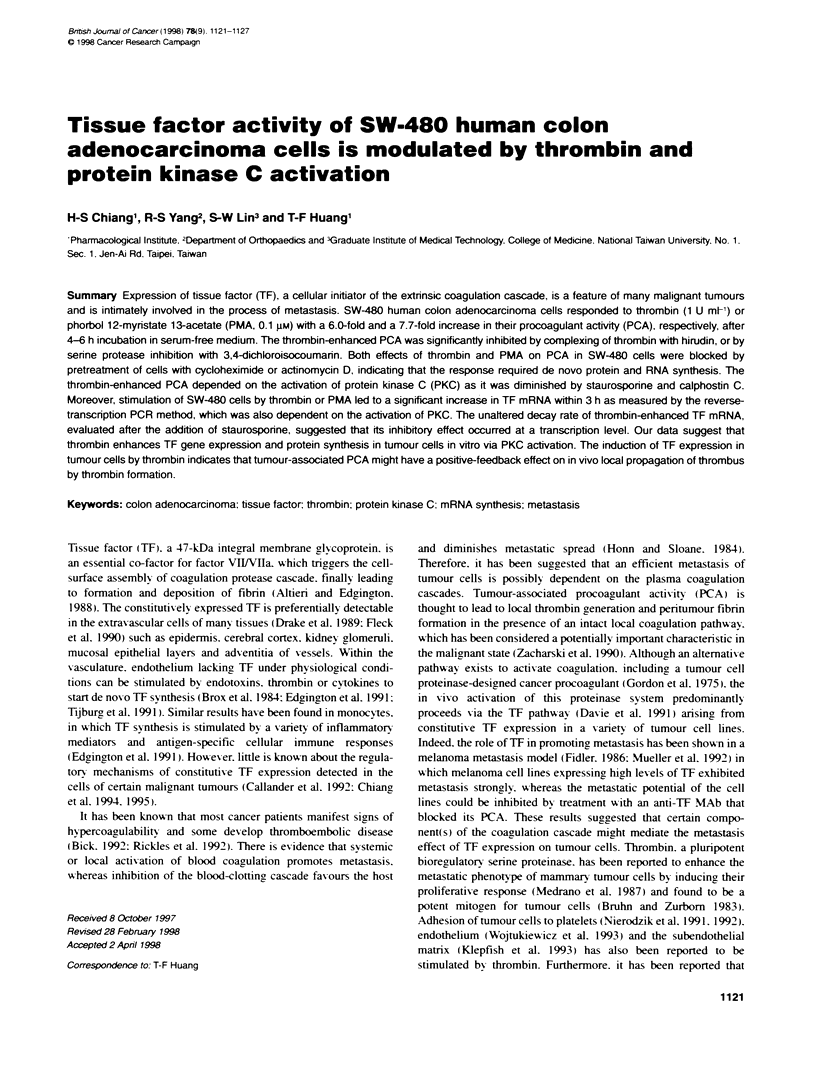

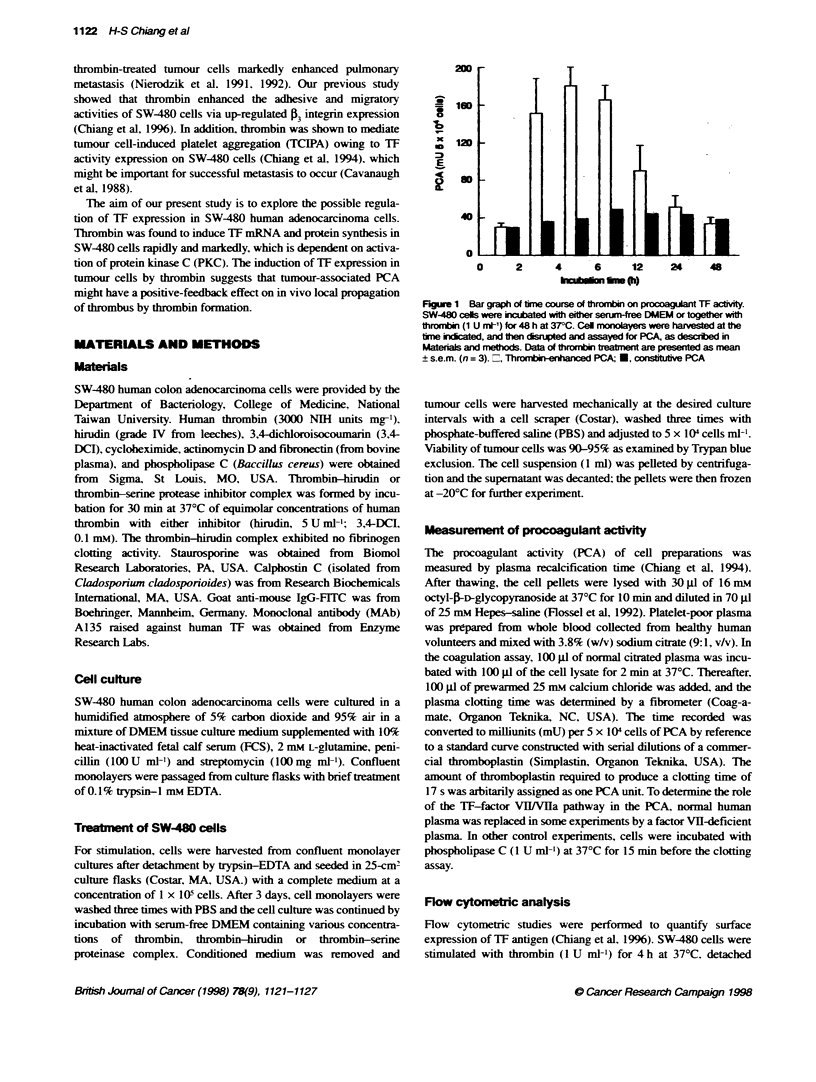

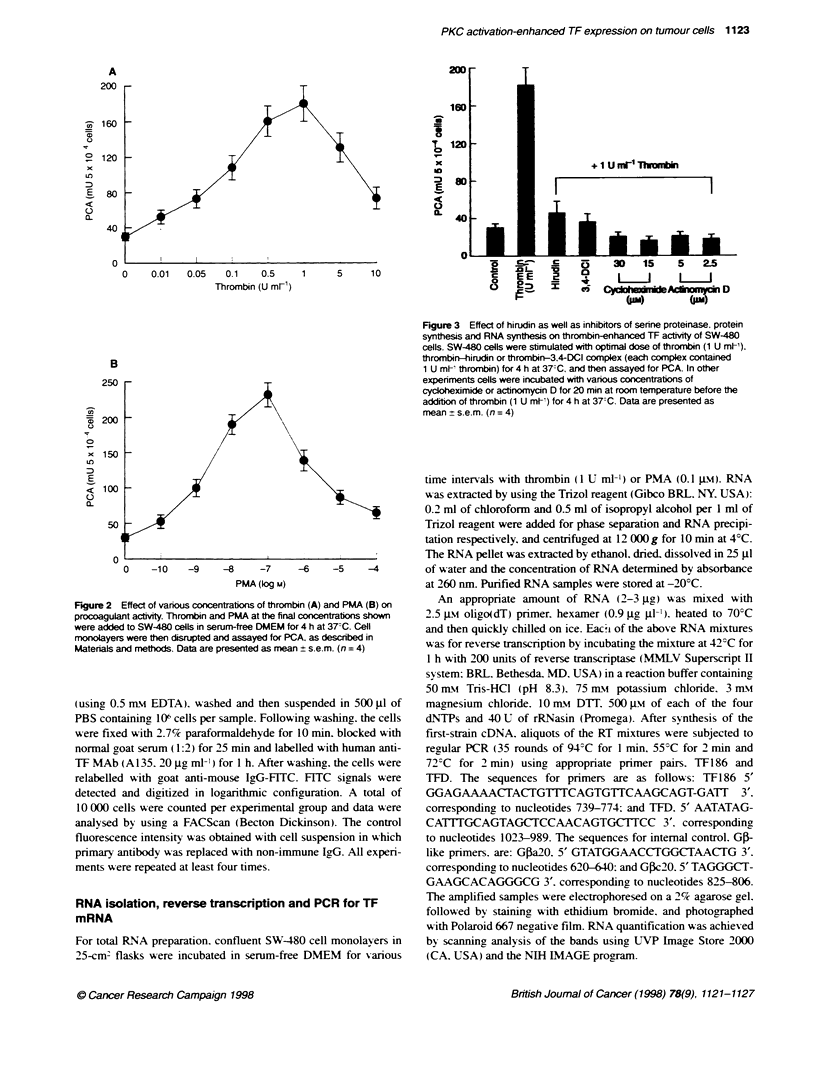

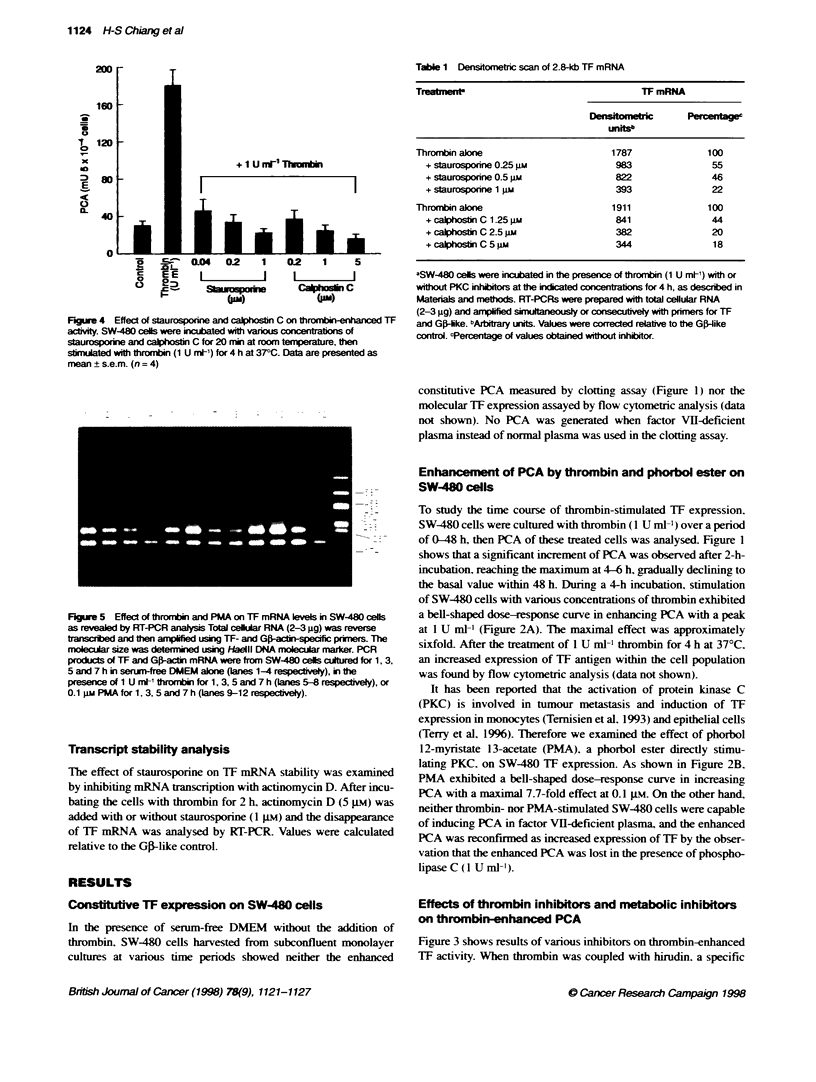

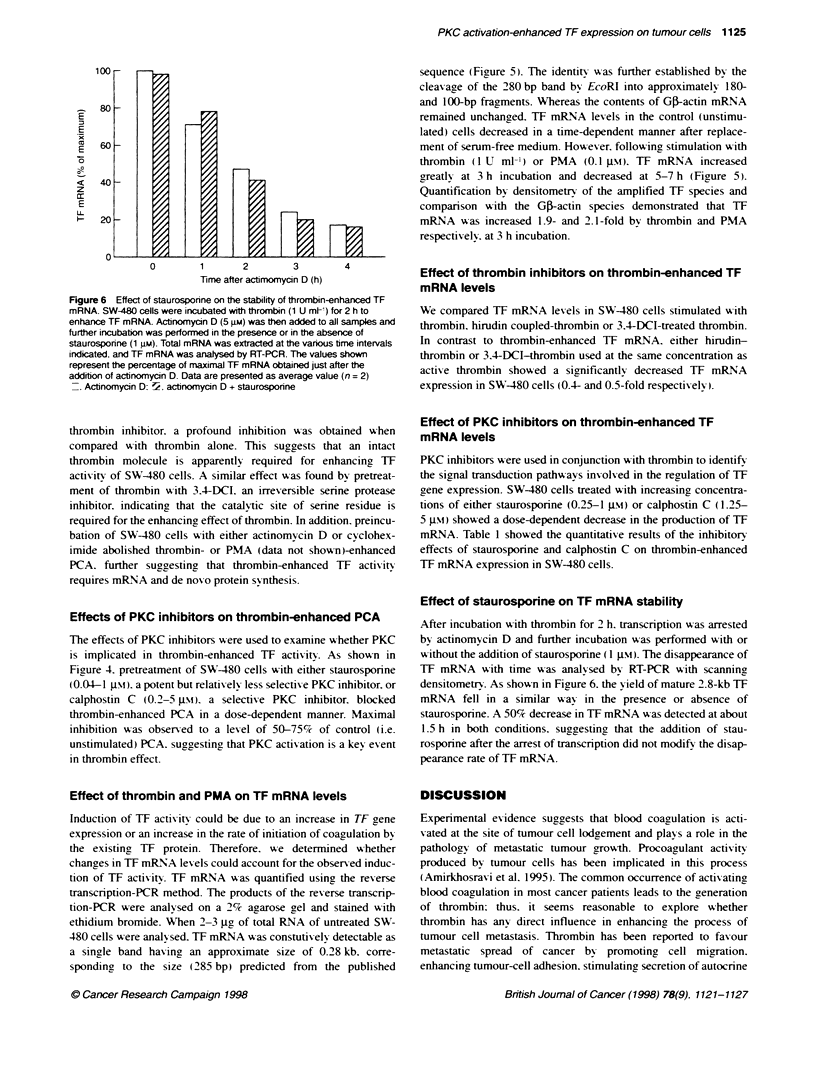

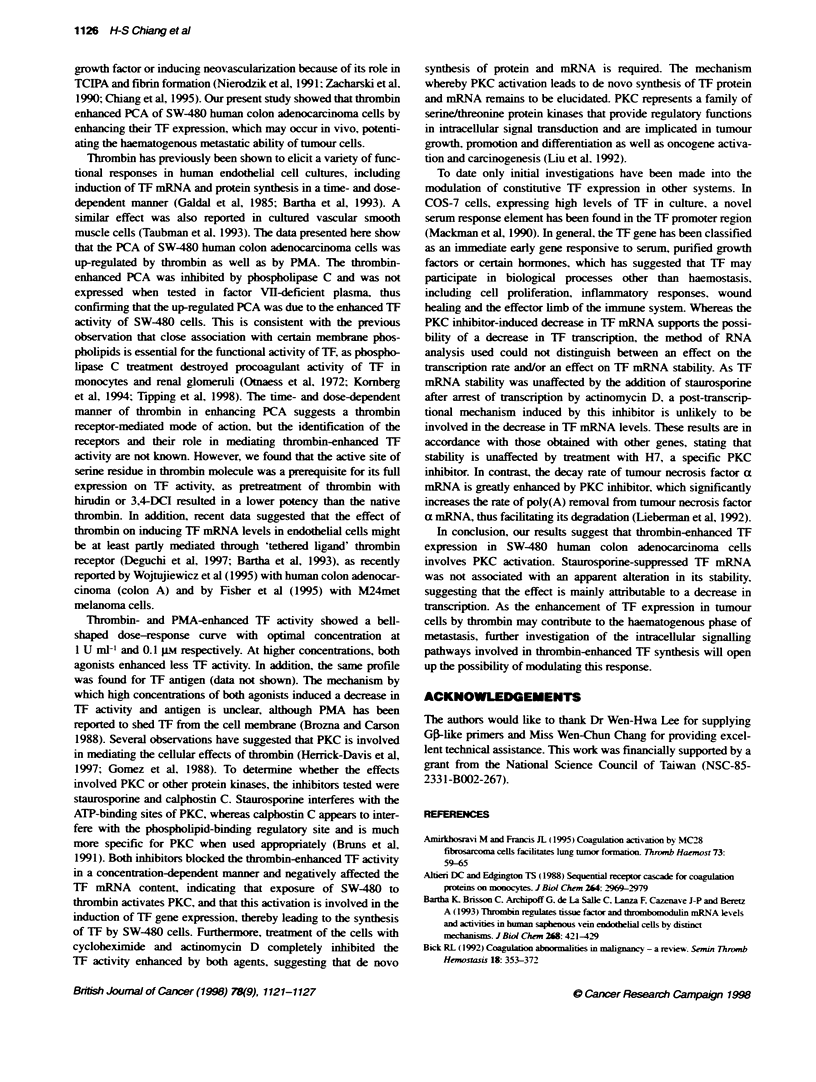

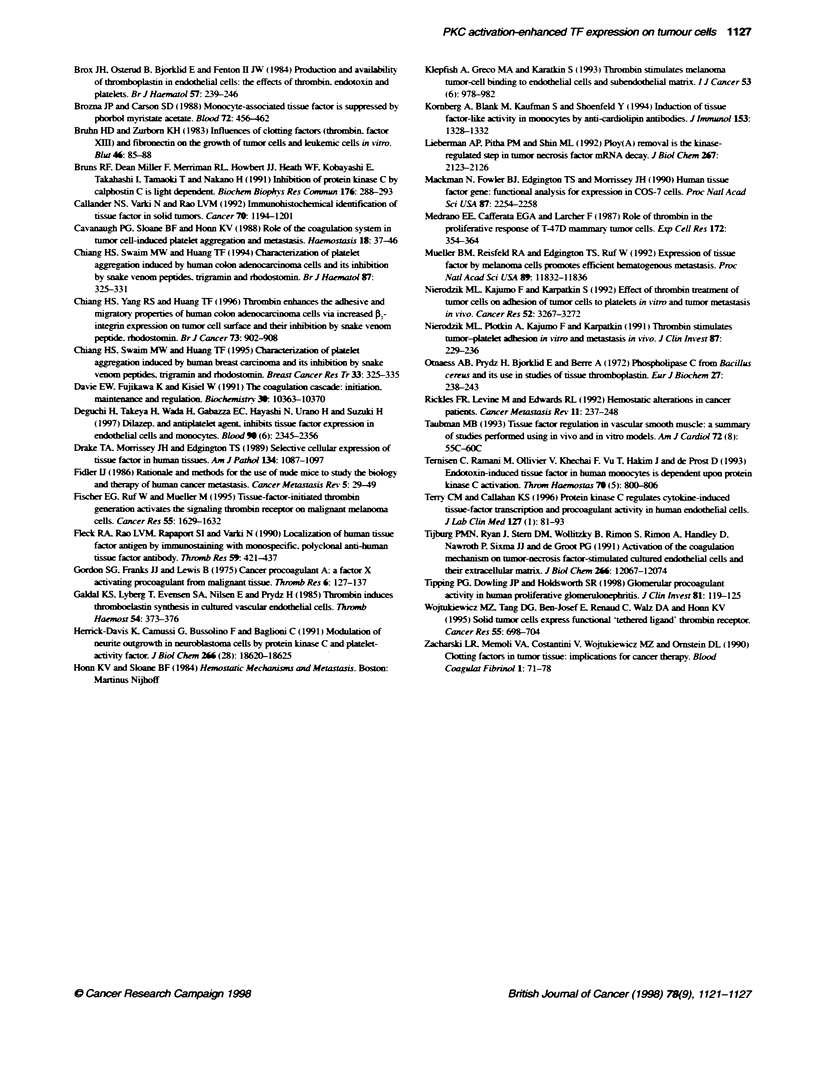


## References

[OCR_00829] Altieri D. C., Edgington T. S. (1989). Sequential receptor cascade for coagulation proteins on monocytes. Constitutive biosynthesis and functional prothrombinase activity of a membrane form of factor V/Va.. J Biol Chem.

[OCR_00824] Amirkhosravi M., Francis J. L. (1995). Coagulation activation by MC28 fibrosarcoma cells facilitates lung tumor formation.. Thromb Haemost.

[OCR_00833] Bartha K., Brisson C., Archipoff G., de la Salle C., Lanza F., Cazenave J. P., Beretz A. (1993). Thrombin regulates tissue factor and thrombomodulin mRNA levels and activities in human saphenous vein endothelial cells by distinct mechanisms.. J Biol Chem.

[OCR_00839] Bick R. L. (1992). Coagulation abnormalities in malignancy: a review.. Semin Thromb Hemost.

[OCR_00847] Brox J. H., Osterud B., Bjørklid E., Fenton J. W. (1984). Production and availability of thromboplastin in endothelial cells: the effects of thrombin, endotoxin and platelets.. Br J Haematol.

[OCR_00852] Brozna J. P., Carson S. D. (1988). Monocyte-associated tissue factor is suppressed by phorbol myristate acetate.. Blood.

[OCR_00856] Bruhn H. D., Zurborn K. H. (1983). Influences of clotting factors (thrombin, factor XIII) and of fibronectin on the growth of tumor cells and leukemic cells in vitro.. Blut.

[OCR_00863] Bruns R. F., Miller F. D., Merriman R. L., Howbert J. J., Heath W. F., Kobayashi E., Takahashi I., Tamaoki T., Nakano H. (1991). Inhibition of protein kinase C by calphostin C is light-dependent.. Biochem Biophys Res Commun.

[OCR_00868] Callander N. S., Varki N., Rao L. V. (1992). Immunohistochemical identification of tissue factor in solid tumors.. Cancer.

[OCR_00870] Cavanaugh P. G., Sloane B. F., Honn K. V. (1988). Role of the coagulation system in tumor-cell-induced platelet aggregation and metastasis.. Haemostasis.

[OCR_00875] Chiang H. S., Swaim M. W., Huang T. F. (1994). Characterization of platelet aggregation induced by human colon adenocarcinoma cells and its inhibition by snake venom peptides, trigramin and rhodostomin.. Br J Haematol.

[OCR_00879] Chiang H. S., Yang R. S., Huang T. F. (1996). Thrombin enhances the adhesion and migration of human colon adenocarcinoma cells via increased beta 3-integrin expression on the tumour cell surface and their inhibition by the snake venom peptide, rhodostomin.. Br J Cancer.

[OCR_00891] Davie E. W., Fujikawa K., Kisiel W. (1991). The coagulation cascade: initiation, maintenance, and regulation.. Biochemistry.

[OCR_00895] Deguchi H., Takeya H., Wada H., Gabazza E. C., Hayashi N., Urano H., Suzuki K. (1997). Dilazep, an antiplatelet agent, inhibits tissue factor expression in endothelial cells and monocytes.. Blood.

[OCR_00900] Drake T. A., Morrissey J. H., Edgington T. S. (1989). Selective cellular expression of tissue factor in human tissues. Implications for disorders of hemostasis and thrombosis.. Am J Pathol.

[OCR_00907] Fischer E. G., Ruf W., Mueller B. M. (1995). Tissue factor-initiated thrombin generation activates the signaling thrombin receptor on malignant melanoma cells.. Cancer Res.

[OCR_00912] Fleck R. A., Rao L. V., Rapaport S. I., Varki N. (1990). Localization of human tissue factor antigen by immunostaining with monospecific, polyclonal anti-human tissue factor antibody.. Thromb Res.

[OCR_00921] Galdal K. S., Lyberg T., Evensen S. A., Nilsen E., Prydz H. (1985). Thrombin induces thromboplastin synthesis in cultured vascular endothelial cells.. Thromb Haemost.

[OCR_00917] Gordon S. G., Franks J. J., Lewis B. (1975). Cancer procoagulant A: a factor X activating procoagulant from malignant tissue.. Thromb Res.

[OCR_00926] Herrick-Davis K., Camussi G., Bussolino F., Baglioni C. (1991). Modulation of neurite outgrowth in neuroblastoma cells by protein kinase C and platelet-activating factor.. J Biol Chem.

[OCR_00935] Klepfish A., Greco M. A., Karpatkin S. (1993). Thrombin stimulates melanoma tumor-cell binding to endothelial cells and subendothelial matrix.. Int J Cancer.

[OCR_00940] Kornberg A., Blank M., Kaufman S., Shoenfeld Y. (1994). Induction of tissue factor-like activity in monocytes by anti-cardiolipin antibodies.. J Immunol.

[OCR_00945] Lieberman A. P., Pitha P. M., Shin M. L. (1992). Poly(A) removal is the kinase-regulated step in tumor necrosis factor mRNA decay.. J Biol Chem.

[OCR_00952] Mackman N., Fowler B. J., Edgington T. S., Morrissey J. H. (1990). Functional analysis of the human tissue factor promoter and induction by serum.. Proc Natl Acad Sci U S A.

[OCR_00957] Medrano E. E., Cafferata E. G., Larcher F. (1987). Role of thrombin in the proliferative response of T-47D mammary tumor cells. Mitogenic action and pleiotropic modifications induced together with epidermal growth factor and insulin.. Exp Cell Res.

[OCR_00962] Mueller B. M., Reisfeld R. A., Edgington T. S., Ruf W. (1992). Expression of tissue factor by melanoma cells promotes efficient hematogenous metastasis.. Proc Natl Acad Sci U S A.

[OCR_00967] Nierodzik M. L., Kajumo F., Karpatkin S. (1992). Effect of thrombin treatment of tumor cells on adhesion of tumor cells to platelets in vitro and tumor metastasis in vivo.. Cancer Res.

[OCR_00970] Nierodzik M. L., Plotkin A., Kajumo F., Karpatkin S. (1991). Thrombin stimulates tumor-platelet adhesion in vitro and metastasis in vivo.. J Clin Invest.

[OCR_00975] Otnaess A. B., Prydz H., Bjorklid E., Berre A. (1972). Phospholipase C from Bacillus cereus and its use in studies of tissue thromboplastin.. Eur J Biochem.

[OCR_00980] Rickles F. R., Levine M., Edwards R. L. (1992). Hemostatic alterations in cancer patients.. Cancer Metastasis Rev.

[OCR_00989] Ternisien C., Ramani M., Ollivier V., Khechai F., Vu T., Hakim J., de Prost D. (1993). Endotoxin-induced tissue factor in human monocytes is dependent upon protein kinase C activation.. Thromb Haemost.

[OCR_00994] Terry C. M., Callahan K. S. (1996). Protein kinase C regulates cytokine-induced tissue factor transcription and procoagulant activity in human endothelial cells.. J Lab Clin Med.

[OCR_01003] Tijburg P. N., Ryan J., Stern D. M., Wollitzky B., Rimon S., Rimon A., Handley D., Nawroth P., Sixma J. J., de Groot P. G. (1991). Activation of the coagulation mechanism on tumor necrosis factor-stimulated cultured endothelial cells and their extracellular matrix. The role of flow and factor IX/IXa.. J Biol Chem.

[OCR_01006] Tipping P. G., Dowling J. P., Holdsworth S. R. (1988). Glomerular procoagulant activity in human proliferative glomerulonephritis.. J Clin Invest.

[OCR_01009] Wojtukiewicz M. Z., Tang D. G., Ben-Josef E., Renaud C., Walz D. A., Honn K. V. (1995). Solid tumor cells express functional "tethered ligand" thrombin receptor.. Cancer Res.

[OCR_01014] Zacharski L. R., Memoli V. A., Costantini V., Wojtukiewicz M. Z., Ornstein D. L. (1990). Clotting factors in tumour tissue: implications for cancer therapy.. Blood Coagul Fibrinolysis.

